# Polymer–Ceramic Hybrid Composites for Lightweight Solar Thermal Collector Absorbers: Thermal Transport, Optical Selectivity, and Durability

**DOI:** 10.3390/polym18060678

**Published:** 2026-03-11

**Authors:** Sachin Kumar Sharma, Reshab Pradhan, Lokesh Kumar Sharma, Yogesh Sharma, Mohit Sharma, Yatendra Pal, Drago Bračun, Damjan Klobčar

**Affiliations:** 1 Surface Science and Tribology Lab, Department of Mechanical Engineering, Shiv Nadar Institution of Eminence, Gautam Buddha Nagar, Greater Noida 201314, India; ss393@snu.edu.in (S.K.S.); rp943@snu.edu.in (R.P.); 2 Department of Physics, GLA University, Mathura 281406, India; lokesh.sharma@gla.ac.in; 3 Department of Physics & Environmental Sciences, Sharda School of Engineering & Science, Sharda University, Greater Noida 201310, India; yogesh.sharma2@sharda.ac.in; 4 Department of Physics and Material Science, Jaypee University, Anoopshahr 203390, India; mohit.sharma@mail.jaypeeu.ac.in; 5 Institute of Education and Research, Mangalayatan University, Aligarh 202146, India; yp.gaur257@gmail.com; 6 Faculty of Mechanical Engineering, University of Ljubljana, Aškerčeva 6, 1000 Ljubljana, Slovenia; drago.bracun@fs.uni-lj.si

**Keywords:** solar thermal collectors, polymer–ceramic hybrid composites, thermally conductive polymers, selective absorber coatings, durability and stagnation resistance

## Abstract

Polymer–ceramic hybrid composites are emerging as attractive candidates for lightweight, corrosion-resistant absorber components in solar thermal collectors; however, their adoption is constrained by the intrinsically low thermal conductivity of polymers, processing-induced anisotropic heat transport, interfacial thermal resistance at tube/laminate joints, and durability challenges under outdoor exposure. This review provides a collector-centered synthesis of polymer–ceramic hybrid materials, emphasizing the translation of composite properties into collector-level outcomes rather than conductivity enhancement alone. A structure–property–performance mapping approach is presented to connect directional thermal conductivity ((k_in-plane), (k_perp)), thermal diffusivity, heat capacity, coefficient of thermal expansion, and service temperature with collector performance parameters such as heat removal effectiveness, overall heat losses, and stagnation behavior. Ceramic fillers (e.g., boron nitride, aluminum nitride, silicon carbide, alumina) are examined for stable conduction-network formation, coating compatibility, and long-term reliability, while carbon fillers (graphite, graphene nanoplatelets, carbon nanotubes) are evaluated for combined heat spreading and solar absorption benefits, with attention to emissivity penalties. Hybrid ceramic–carbon architectures and multilayer absorber designs are identified as the most promising routes to balance thermal transport, optical selectivity (high solar absorptance and low thermal emittance), manufacturability, and durability under UV, humidity, and thermal cycling.

## 1. Introduction

Solar thermal collectors are among the most efficient technologies for converting solar irradiation into usable heat for domestic hot water, space heating, industrial process heat, and emerging applications such as low-temperature desalination and absorption cooling. Conventional flat-plate and evacuated tube collectors typically employ metallic absorber components, most commonly copper or aluminum, because of their high thermal conductivity and effective integration with heat exchangers [1,2,3]. However, metal absorbers increase system weight and cost and may suffer from corrosion during long-term outdoor operation, which has motivated the exploration of lightweight and corrosion-resistant alternatives [4,5]. Polymer-based materials have therefore attracted increasing attention for solar thermal systems operating in low-to-moderate temperature ranges (generally below 120–150 °C). Their low density, design flexibility, corrosion resistance, and compatibility with scalable manufacturing methods such as extrusion, compression molding, and roll-to-roll processing make them attractive candidates for lightweight collector components [6,7]. A key limitation, however, is the intrinsically low thermal conductivity of most polymers (typically ~0.1–0.5 W·m^−1^·K^−1^), which restricts heat spreading within absorber structures and can lead to increased temperature gradients during operation [8]. These gradients may reduce heat removal efficiency and accelerate degradation processes such as creep, thermo-oxidative aging, and coating or interface delamination.

To address these challenges, polymer–ceramic hybrid composites have emerged as promising materials for next-generation lightweight solar thermal absorbers [9,10,11]. Incorporating thermally conductive ceramic fillers (e.g., Al_2_O_3_, SiC, AlN, BN, TiO_2_) and carbon-based reinforcements (e.g., graphene, carbon nanotubes, and graphite) into polymer matrices can create conductive heat-transfer pathways while preserving the inherent advantages of polymers, including corrosion resistance and manufacturing flexibility [12,13]. In addition to improving thermal transport, these fillers can enhance mechanical stiffness, reduce the coefficient of thermal expansion (CTE), and improve resistance to UV exposure and moisture, all of which are important for long-term outdoor durability in solar collector environments.

Beyond thermal transport, absorber materials must satisfy multiple performance requirements that determine collector efficiency and long-term reliability. In solar thermal systems, performance is governed by the balance between absorbed solar energy, effective heat removal, and thermal losses [14]. Consequently, absorber materials must exhibit suitable optical properties, including high solar absorptance (*α*) and low thermal emittance (ε), either intrinsically or through compatibility with selective surface coatings. In addition, real collector operation involves thermal cycling, humidity exposure, freeze–thaw conditions, and stagnation events that may exceed normal operating temperatures [15]. These conditions can degrade polymer composites through mechanisms such as hydrolytic aging, UV-induced chain scission, and filler–matrix interfacial weakening, which may gradually reduce thermal transport performance and structural stability [16]. For this reason, polymer–ceramic hybrid materials intended for solar collectors must be evaluated using collector-relevant metrics and durability testing protocols rather than thermal conductivity values alone [17]. Although thermally conductive polymer composites have been widely studied for electronic thermal management, their application in solar thermal collectors introduces additional constraints related to optical selectivity, coating adhesion, environmental durability, and performance translation from material properties to collector efficiency [18]. Furthermore, reported thermal conductivities vary widely across the literature due to differences in filler morphology, loading fraction, dispersion quality, orientation, and processing methods. Comparisons between studies are often complicated by inconsistent reporting of measurement direction (in-plane versus through-plane conductivity), temperature conditions, and testing methodologies. These inconsistencies highlight the need for a systematic synthesis that evaluates polymer–ceramic hybrid materials using collector-relevant performance criteria.

This review presents a collector-oriented assessment of polymer–ceramic hybrid composites for lightweight solar thermal absorber components. Rather than focusing solely on thermal conductivity enhancement, the analysis integrates thermal transport, optical selectivity, durability under outdoor exposure, and manufacturing feasibility to evaluate the practical suitability of these materials for collector applications. This review establishes collector-relevant material property targets including thermal conductivity, coefficient of thermal expansion, thermal stability limits (Tg/Tm), optical absorptance and emittance (*α*/ε), and durability thresholds and examines how these parameters influence collector design and long-term performance. The manuscript is organized as follows: Section 2 outlines collector requirements and key material property targets; Section 3 discusses candidate polymer matrices; Section 4 reviews ceramic and carbon fillers; Section 5 examines structure–property–performance relationships; Section 6 summarizes fabrication and manufacturing considerations; Section 7 addresses durability and outdoor aging; and Section 8 provides a comparative synthesis of collector-ready composite strategies.

## 2. Solar Thermal Collector Requirements and Material Property Targets

The performance of solar thermal collectors is governed by the balance between absorbed solar energy and thermal losses, commonly described using the Hottel–Whillier model for flat-plate collectors [19]. Collector efficiency (η) can be expressed as follows:

$$\eta =F_{R}(\tau \alpha )-F_{R}U_{L}(T_{in}-T_{a})/G,$$

where $F_{R}$ is the heat removal factor, $\left(\tau \alpha \right)$ represents the effective optical gain, $U_{L}$ is the overall heat loss coefficient, $T_{in}$ and $T_{a}$ denote the inlet and ambient temperatures, and G is the solar irradiance [20,21]. Although this formulation is traditionally applied to metallic absorber plates, it also provides a useful framework for evaluating polymer–ceramic hybrid absorbers. In such systems, the thermal and mechanical properties of the absorber material influence heat removal effectiveness, temperature gradients within the absorber, and long-term operational stability.

A primary requirement for polymer–ceramic hybrid absorber materials is adequate thermal transport to ensure efficient heat spreading and transfer to the working fluid [22]. Conductive fillers such as boron nitride, aluminum nitride, alumina, silicon carbide, graphite, graphene, and carbon nanotubes are commonly incorporated into polymer matrices to establish thermally conductive pathways. However, composite absorbers frequently exhibit anisotropic thermal transport due to filler alignment and processing-induced orientation effects [23,24]. From a collector design perspective, in-plane thermal conductivity governs lateral heat spreading and uniform surface temperature distribution across the absorber plate [25,26], whereas through-plane conductivity controls heat transfer into flow channels or bonded tubes. Consequently, collector-grade hybrid absorbers typically aim for in-plane conductivity values of ≥5 W·m^−1^·K^−1^ (preferably higher) and through-plane conductivity of approximately 1–2 W·m^−1^·K^−1^.

**Figure 1:**
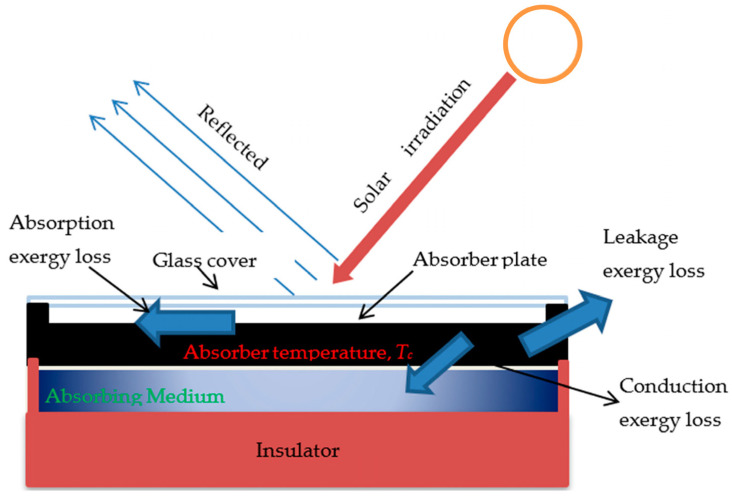
Schematic representation of exergy loss mechanisms in a flat-plate solar collector, including absorption exergy loss (solar radiation → absorber), leakage exergy loss (absorber → ambient), and conduction exergy loss (absorber → working medium) [34].

Thermal transport properties should also be considered alongside thermal diffusivity and heat capacity, particularly during transient operating conditions. Thermal diffusivity $\left(\alpha =k/\rho C_{p}\right)$ determines how rapidly heat spreads within the absorber during fluctuations in solar irradiance or fluid flow [27]. While filler incorporation can enhance conductivity, it may also affect density and heat capacity, influencing overall heat-transfer behavior [28]. Balanced improvements in conductivity and diffusivity are therefore desirable to minimize hot-spot formation and maintain stable absorber temperatures during operation. Thermal stability is another critical requirement, particularly during stagnation events when solar irradiation remains high but heat removal is limited or interrupted. Under these conditions, absorber temperatures may exceed normal operating levels, potentially causing polymer softening, creep deformation, or dimensional distortion [29]. Elevated temperatures can also accelerate thermo-oxidative degradation and interfacial damage between the polymer matrix, fillers, coatings, and bonding layers [30]. For this reason, polymer–ceramic hybrid absorbers should possess sufficient thermal stability, with glass transition or melting temperatures (Tg/Tm) exceeding the maximum expected operating temperatures [31]. In addition, controlling the coefficient of thermal expansion (CTE) is essential because polymer absorber plates are frequently bonded to metal tubes or coated with selective surfaces. Reducing CTE through filler incorporation can improve interfacial stability and mitigate delamination during thermal cycling [32]. Optical performance is equally important because collector efficiency strongly depends on absorber solar absorptance ($\alpha_{solar}$) and thermal emittance ($\varepsilon_{thermal}$). High absorptance (typically *α* ≥ 0.90) maximizes solar energy capture, while low emittance (ε ≤ 0.20) suppresses radiative heat losses at elevated temperatures [33,34]. Since polymers generally lack intrinsic optical selectivity, absorber surfaces usually require compatible selective coatings, high-absorptance paints, or engineered surface structures. Ceramic fillers may improve coating adhesion and dimensional stability, whereas carbon fillers can enhance broadband solar absorption but must be carefully optimized to avoid excessive thermal emissivity [33]. Collector performance can also be interpreted from an exergy perspective, which highlights where useful energy is degraded within the system. As illustrated in Figure 1, irreversibilities arise from absorption losses during conversion of solar radiation to absorber heat, leakage losses to the ambient environment, and conduction losses during heat transfer from the absorber plate to the working fluid [34]. Reducing temperature gradients within the absorber and minimizing heat leakage pathways are therefore important strategies for improving overall collector performance.

**Figure 2:**
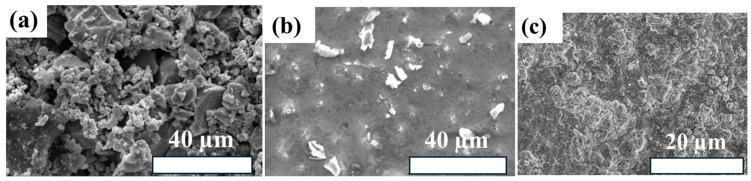
(**a**–**c**) SEM micrographs of multi-scale cermet solar selective absorber coatings showing hierarchical granular morphology and dense microstructural packing, which governs light trapping, oxidation resistance, and thermal-cycling stability at elevated temperatures [35].

Microstructural integrity plays a key role in maintaining the optical selectivity and thermal stability of cermet absorber coatings. As shown in Figure 2a–c, the coating surface exhibits a hierarchical granular morphology composed of densely packed ceramic and metallic domains with finer dispersed features, characteristic of multi-scale cermet structures rather than uniform single-phase films [35]. This microstructural organization enhances photothermal performance by promoting light trapping and enabling controlled effective refractive index gradients. At the same time, the dense and well-bonded structure improves high-temperature stability by limiting crack propagation and suppressing microstructural coarsening. Such features also reduce oxygen diffusion and interfacial degradation during thermal cycling, thereby helping to maintain stable absorptance and emissivity under prolonged high-temperature operation.

Durability under outdoor conditions represents another important requirement for absorber materials. Solar collectors are typically expected to operate for decades under ultraviolet radiation, humidity cycling, temperature fluctuations, and environmental exposure. These conditions may induce polymer degradation, interfacial weakening, and coating instability if materials are not properly designed. Consequently, polymer–ceramic hybrid absorbers should demonstrate stable thermal conductivity, maintained coating adhesion, and resistance to filler–matrix debonding after accelerated aging tests such as UV exposure, humidity aging, thermal cycling, and stagnation simulations [35,36,37]. To facilitate practical material selection for solar thermal collectors, the requirements discussed above can be translated into representative property targets for polymer–ceramic hybrid absorber components. These targets reflect the combined thermal, optical, and durability constraints imposed by collector operation. In particular, high in-plane conductivity promotes effective heat spreading across absorber surfaces, while adequate through-plane conductivity supports heat transfer into fluid channels or bonded tubes, thereby influencing the heat removal factor and overall collector efficiency. At the same time, controlled thermal expansion, high service temperature, and environmental stability ensure reliable operation under long-term outdoor exposure. Table 1 summarizes recommended property ranges and their relevance to collector-level performance, providing a practical framework for evaluating polymer–ceramic hybrid materials intended for solar thermal absorber applications.

### Translation of Material Properties to Collector Performance

**Figure 3:**
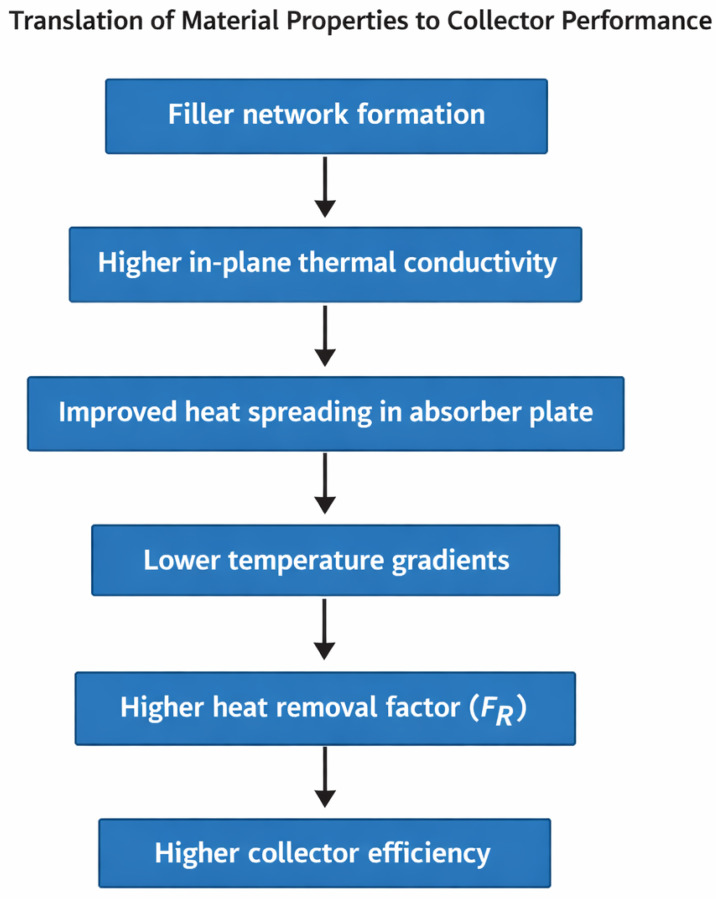
Schematic representation linking polymer–ceramic composite microstructure to solar collector performance. Enhanced filler networks increase in-plane thermal conductivity, improve heat spreading in the absorber plate, reduce temperature gradients, and increase the heat removal factor ($F_{R}$), resulting in higher collector efficiency.

The practical value of polymer–ceramic hybrid absorbers can only be fully assessed by linking composite material properties with collector-level performance metrics. Within the Hottel–Whillier framework, solar collector efficiency depends on optical gain, heat removal effectiveness, and thermal losses [36]. Consequently, improvements in composite thermal transport properties must ultimately translate into enhanced heat removal and reduced temperature gradients within the absorber structure. Thermal conductivity plays a particularly important role in determining absorber temperature distribution and heat transfer to the working fluid [37,38]. Increased in-plane thermal conductivity improves lateral heat spreading across the absorber plate, reducing localized hot spots and promoting uniform temperature distribution under non-uniform solar irradiation. Improved temperature uniformity directly contributes to higher heat removal effectiveness by enabling more efficient transfer of absorbed heat to fluid channels or bonded tubes [39]. In contrast, through-plane thermal conductivity governs heat transfer from the absorber surface toward the fluid pathways and therefore directly influences the heat removal factor in flat-plate collectors. Thermo-mechanical properties also influence collector performance and long-term reliability [41]. Reduced coefficients of thermal expansion and improved composite stiffness help maintain stable tube–absorber interfaces and prevent coating delamination during thermal cycling [42]. These characteristics are particularly important under stagnation conditions, where absorber temperatures may temporarily exceed normal operating limits. Materials with improved thermal transport and dimensional stability therefore contribute to both higher collector efficiency and improved durability during long-term outdoor operation [43,44,45]. The relationship between composite microstructure and collector performance can be conceptually represented as a materials-to-system translation pathway (Figure 3). In practical absorber designs, even moderate increases in composite conductivity can significantly influence temperature uniformity and heat transfer. For instance, increasing absorber plate thermal conductivity from approximately 0.5 Wm^−1^ K^−1^ (typical polymer) to around 5 Wm^−1^ K^−1^ (polymer–ceramic composite) can reduce temperature gradients across polymer absorber plates by more than 50%, thereby improving heat removal effectiveness and reducing stagnation-induced degradation risks [44,45]. Such improvements demonstrate why thermal conductivity targets, presented earlier in Table 1, are directly relevant to collector-level design and performance.

## 3. Polymer Matrices for Collector-Grade Polymer–Ceramic Hybrid Absorbers

The polymer matrix plays a central role in determining the service temperature, thermo-mechanical stability, durability, and processing compatibility of polymer–ceramic hybrid absorber components. While ceramic and carbon fillers establish thermally conductive pathways, the matrix governs filler dispersion, interfacial adhesion, and resistance to environmental degradation. In solar thermal collectors, the matrix must tolerate prolonged exposure to elevated temperatures and outdoor conditions without excessive creep, embrittlement, or interfacial deterioration [49,50]. Consequently, matrix selection represents a critical design constraint that determines whether conductivity enhancements achieved through fillers translate into reliable absorber performance [51].

Polyolefins such as polyethylene (PE) and polypropylene (PP) have attracted interest due to their low cost, low density, and compatibility with large-scale extrusion processing. These characteristics make them attractive for low-temperature polymer absorber concepts and cost-sensitive collector designs. However, their relatively limited thermal stability and susceptibility to creep deformation at elevated temperatures restrict their use in demanding collector environments [50,51]. In addition, UV-induced oxidation and relatively high coefficients of thermal expansion can compromise long-term dimensional stability unless stabilization strategies or protective encapsulation are applied. As a result, PE- and PP-based composites are generally most suitable for low-temperature collectors where stagnation conditions are controlled or mechanical stresses are minimized.

Engineering polymers such as polyamide (PA), polycarbonate (PC), polyphenylene oxide (PPO), and acrylonitrile butadiene styrene (ABS) provide improved mechanical strength and higher operating temperature limits compared with commodity polymers. These materials can serve as intermediate candidates for moderate-temperature collector components. Nevertheless, challenges such as moisture absorption, hydrolytic degradation (particularly for PA), and UV sensitivity must be addressed through stabilizers, coatings, or material modifications to ensure long-term durability [52,53]. For absorber plates or channel structures requiring dimensional stability, these polymers may offer a useful balance between cost, processability, and thermal capability when properly protected. High-temperature polymers including polyphenylene sulfide (PPS), polyether ether ketone (PEEK), polyetherimide (PEI), polyimide (PI), and high-temperature fluoropolymers represent the most promising matrix class for collector applications where stagnation tolerance and long-term durability are critical [52]. PPS and PEEK in particular exhibit excellent chemical resistance [54], high thermal stability, and reduced creep susceptibility relative to commodity polymers [55]. These characteristics make them suitable for absorber components exposed to elevated temperatures and repeated thermal cycling. Although these materials increase system cost, they enable hybrid absorbers capable of operating at higher temperatures with improved structural reliability. Importantly, their superior thermal stability also helps maintain filler network integrity during thermal cycling, preventing loss of conductivity caused by matrix softening or interfacial debonding.

Thermosetting matrices, including epoxy, phenolic, and silicone-based resins, provide an additional material platform for polymer–ceramic hybrid absorbers [56]. Thermosets generally offer good dimensional stability, strong adhesion to coatings and fillers, and improved resistance to creep at elevated temperatures [57]. However, brittleness and microcrack formation under repeated thermal cycling may limit long-term durability unless toughening strategies are implemented [58]. Silicone-based matrices are particularly attractive for absorber encapsulation or coating-compatible substrates because of their high thermal stability and UV resistance, although their lower mechanical stiffness can restrict structural applications unless reinforced. From a manufacturing perspective, thermosets are typically less compatible with high-volume extrusion processes than thermoplastics, but they remain useful for specialized absorber architectures or multilayer composite designs.

Overall, polymer matrix selection for solar thermal collectors must be guided by collector-specific constraints rather than generic composite design considerations. The most important selection criteria include: (i) service temperature and stagnation tolerance [59]; (ii) creep resistance and thermo-mechanical stability during thermal cycling [60]; (iii) coefficient of thermal expansion and compatibility with metallic tubes or selective coatings [61]; (iv) resistance to UV radiation and moisture exposure [62]; and (v) manufacturability in absorber geometries suitable for collector integration [63]. Table 2 summarizes commonly used polymer matrices and compares their thermal capability, durability limitations, and suitability for polymer–ceramic hybrid absorber components. This overview provides practical guidance for selecting matrix systems capable of maintaining filler-network integrity and long-term reliability in solar collector environments.

## 4. Ceramic and Carbon Fillers for Collector-Grade Polymer–Ceramic Hybrid Absorbers

Thermally conductive fillers are essential for enabling polymer-based absorber components in solar thermal collectors, where unfilled polymers generally exhibit insufficient thermal conductivity for efficient heat spreading and transfer into flow channels or bonded heat exchangers. In collector applications, filler selection must consider not only conductivity enhancement but also optical behavior, stability under stagnation conditions, compatibility with selective coatings, and long-term environmental durability. Unlike electronic thermal management systems, solar collectors impose coupled thermal–optical constraints, meaning that fillers that improve thermal transport may also influence solar absorptance, thermal emittance, surface roughness, and coating adhesion. Consequently, filler selection for polymer–ceramic hybrid absorbers must be evaluated within a collector-centered performance framework.

Ceramic fillers such as hexagonal boron nitride (h-BN) [77], boron nitride nanosheets (BNNS) [78], aluminum nitride (AlN) [79], silicon carbide (SiC) [80], alumina (Al_2_O_3_) [81], titanium dioxide (TiO_2_) [82], and zinc oxide (ZnO) [83] are widely studied for thermally conductive polymer composites due to their thermal stability, chemical resistance, and mechanical reinforcement capability. Among these, boron nitride is particularly attractive for solar absorber composites because it combines high intrinsic thermal conductivity with electrical insulation and strong resistance to oxidation. Platelet-shaped h-BN and BN nanosheets also tend to align during processing, enabling enhanced in-plane heat spreading that helps reduce temperature non-uniformity and local hot spots in absorber plates. Aluminum nitride and silicon carbide offer similarly high thermal conductivity and mechanical strength [84,85], although AlN requires moisture control in humid environments and SiC can increase composite density when used at high loading fractions. Alumina is more economical and chemically robust but generally provides smaller conductivity improvements unless high filler fractions are used. TiO_2_ and ZnO are typically employed as functional additives rather than primary thermal fillers, contributing to UV shielding and optical tuning that improves the long-term stability of polymer matrices.

Carbon-based fillers, including graphite, graphene nanoplatelets (GNP), carbon nanotubes (CNTs), and carbon black, provide a complementary approach to enhancing absorber performance. In addition to improving thermal transport, these materials can significantly increase broadband solar absorption [86,87], which is beneficial for absorber surfaces requiring high solar absorptance. However, carbon fillers may also increase thermal emittance, potentially raising radiative heat losses at elevated operating temperatures. Graphite and graphene platelets are particularly effective for enhancing in-plane thermal conductivity through aligned conductive networks, enabling efficient heat spreading across absorber surfaces. Carbon nanotubes can achieve percolated conductive networks at relatively low loading fractions due to their high aspect ratio [88], although dispersion challenges and increased processing viscosity may limit large-scale applications [89]. Carbon black is commonly used as a low-cost absorber pigment because of its strong optical absorption [90,91], although its contribution to thermal conductivity is relatively modest compared with platelet or nanotube fillers [92].

Hybrid filler architectures that combine ceramic and carbon components represent a promising strategy for balancing thermal and optical performance in polymer–ceramic absorbers. Ceramic-dominant networks such as BN-based systems provide stable heat conduction and good coating compatibility, while controlled fractions of carbon fillers can enhance solar absorptance without excessively increasing thermal emittance [93,94]. Hybrid networks can also reduce interfacial thermal resistance and improve conduction pathways through multi-scale particle packing, where larger ceramic fillers form the primary conduction framework and nanoscale carbon fillers bridge gaps between particles. Such designs are particularly attractive for collectors operating at moderate to high temperatures, where both thermal transport and optical selectivity must be carefully balanced.

Filler morphology also plays an important role in determining conduction pathways and anisotropic thermal transport. Platelet fillers such as BN, graphite, and graphene tend to align during extrusion or compression processing, producing composites with high in-plane conductivity but relatively limited through-plane heat transfer. Fiber-like fillers such as CNTs can promote three-dimensional conductive networks but require careful dispersion to prevent aggregation. In contrast, spherical or irregular ceramic particles such as Al_2_O_3_, TiO_2_, and ZnO generally produce more isotropic thermal transport but often require higher loading fractions to achieve collector-relevant conductivity levels [95,96]. These microstructural characteristics directly influence absorber performance and must therefore be considered alongside intrinsic filler conductivity. To facilitate collector-oriented material selection, Table 3 summarizes the principal ceramic and carbon fillers used in polymer–ceramic hybrid absorbers, highlighting their thermal conductivity potential, anisotropy behavior, optical influence, durability considerations, and processing characteristics. The comparison indicates that BN-based fillers generally offer the most favorable combination of thermal performance, chemical stability, and coating compatibility, while carbon fillers provide valuable optical absorption but require careful optimization to control emissivity. Hybrid ceramic–carbon architectures therefore represent a particularly promising pathway for achieving balanced thermal and optical performance in lightweight solar thermal absorber materials.

Overall, the comparison in Table 3 highlights that no single filler simultaneously optimizes thermal transport, optical response, and durability for solar thermal collectors. Ceramic fillers, particularly BN-based systems, generally provide the most stable thermal conduction networks and strong compatibility with selective absorber coatings, making them well suited for maintaining long-term collector reliability. Carbon fillers offer strong solar absorption and efficient heat spreading but require careful control of loading fraction and surface design to avoid excessive thermal emittance. Consequently, hybrid ceramic–carbon architectures are increasingly viewed as the most promising approach for collector-grade polymer–ceramic composites, as they allow for thermal conductivity, solar absorptance, and emissivity to be tuned simultaneously while maintaining durability under outdoor operating conditions.

## 5. Structure–Property–Performance Relationships in Polymer–Ceramic Hybrid Absorbers

The thermal and functional performance of polymer–ceramic hybrid absorbers in solar thermal collectors is governed not only by filler selection but also by how fillers are distributed, oriented, and coupled to the polymer matrix across multiple length scales. In collector-grade absorber components, composite microstructure determines the formation of thermally conductive pathways and therefore controls the extent to which improvements in intrinsic composite properties translate into collector-level performance gains, reduce stagnation risk, and improved long-term durability [107,108]. Unlike metallic absorbers, polymer-based absorber plates or channel structures are often limited by internal conduction resistance and localized temperature gradients, making microstructure design critical for effective heat spreading and thermal stability during operation [108].

Thermal transport in polymer–ceramic hybrids is enhanced through several mechanisms, including the formation of percolated filler networks, reduction in phonon scattering through high-conductivity pathways, and improved interfacial thermal coupling between the filler and polymer matrix. At low filler loading, heat transfer is dominated by the polymer matrix and isolated fillers provide only modest conductivity gains. As filler concentration increases, particle–particle contacts become more frequent and interconnected conduction networks emerge, producing a rapid rise in thermal conductivity once a percolation-like threshold is reached [109]. In collector applications, this transition is particularly important because conductivity must exceed practical thresholds before meaningful improvements in absorber heat spreading are realized. However, excessively high filler loading can increase melt viscosity, reduce processing quality, and introduce brittleness or interfacial crack initiation under thermal cycling [110]. Consequently, collector-oriented composite design requires balancing thermal conductivity enhancement with manufacturability, mechanical stability, and environmental durability [111].

**Figure 4:**
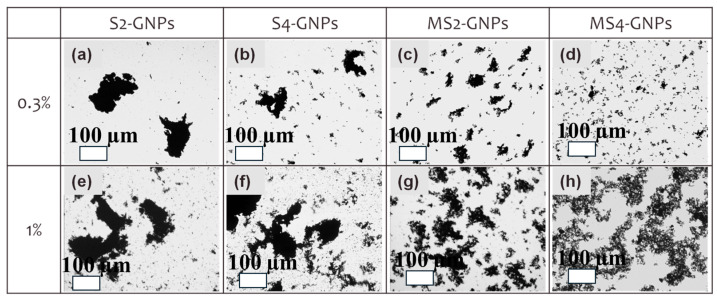
(**a**–**h**) Optical micrographs showing dispersion and agglomeration states of graphene nanoplatelets (GNPs) in epoxy composites at 0.3 wt.% and 1 wt.% loading for different processing routes. S2-GNPs and S4-GNPs correspond to sonication-only processing (2 and 4 h), whereas MS2-GNPs and MS4-GNPs denote bead-milled followed by sonicated samples. The images illustrate the transition from isolated platelet clusters to more interconnected conductive networks with increasing filler loading [112].

A major factor limiting thermal transport in polymer composites is interfacial thermal resistance, often referred to as Kapitza resistance, which arises from phonon mismatch between filler particles and the surrounding polymer matrix as well as imperfect interfacial bonding [109,110]. Even fillers possessing very high intrinsic thermal conductivity may provide limited composite improvement if heat transfer across the filler–matrix interface is inefficient [111,112]. Figure 4a–h illustrates the influence of dispersion and agglomeration on conductive network formation in graphene nanoplatelet (GNP)–filled epoxy composites. At low loading (0.3 wt.%), large GNP variants exhibit pronounced micron-scale clustering with isolated agglomerates, whereas mechanically processed or milled GNPs display a more distributed platelet population. At higher loading (1 wt.%), the probability of platelet–platelet contact increases, initiating the formation of conductive pathways [112]. However, excessive clustering can create interfacial voids and weak interphase regions that limit stress transfer and create localized heat-flow bottlenecks. This dispersion-controlled transition from isolated clusters to interconnected platelet networks represents a key microstructural driver governing both thermal and mechanical performance in platelet-filled composites [111,112].

**Figure 5:**
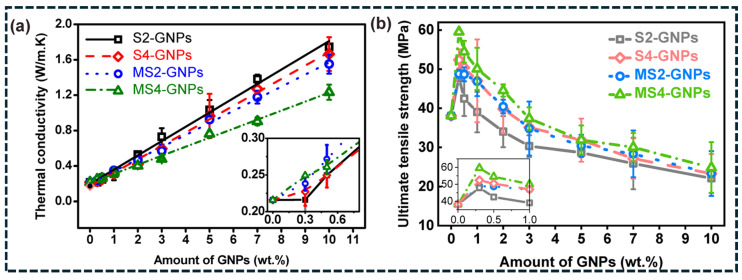
Thermal and mechanical behavior of GNP-filled epoxy composites as a function of GNP loading. (**a**) Thermal conductivity increase associated with platelet network formation and reduced polymer-dominated heat transport. (**b**) Tensile strength variation showing the trade-off between enhanced conductivity and mechanical degradation at higher filler loadings due to agglomeration and interfacial defects [112].

Consistent with this mechanism, Figure 5a shows a monotonic increase in thermal conductivity with increasing GNP fraction, driven by percolation-assisted platelet networking and reduced polymer-dominated heat-flow length. The magnitude of conductivity enhancement depends strongly on platelet morphology and processing history. Larger or less-fragmented platelets produced through sonication-only processing provide higher conductivity at equivalent loading due to their higher aspect ratio and larger contact area, which lowers junction resistance between platelets [112]. In contrast, milling-induced fragmentation improves dispersion but increases the number of platelets–platelet interfaces and therefore raises cumulative interfacial resistance. At the same time, Figure 5b demonstrates that tensile strength peaks at low GNP concentrations but decreases at higher loading levels due to agglomeration-induced stress concentration and interphase debonding. These results highlight the trade-off between maximizing conductive pathway connectivity and maintaining mechanical integrity under thermal cycling conditions [112].

**Figure 6:**
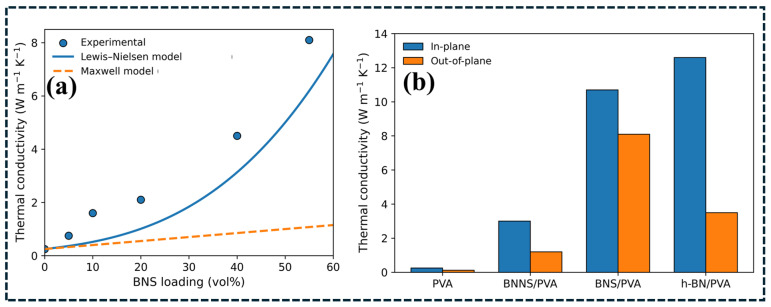
Thermal conductivity behavior of boron nitride sheet (BNS) filled PVA composites. (**a**) Thermal conductivity as a function of BNS loading compared with effective-medium predictions (Lewis–Nielsen and Maxwell models), illustrating the transition from dilute dispersion to network-assisted heat transport. (**b**) Comparison of in-plane and out-of-plane conductivity demonstrating strong anisotropy resulting from preferential alignment of two-dimensional fillers [115].

The influence of filler connectivity and anisotropic heat transport is further illustrated in Figure 6, which presents thermal conductivity trends for BN-based composites. As shown in Figure 6a, the thermal conductivity of BNS/PVA composites increases sharply with rising boron nitride sheet loading, deviating from the Maxwell dilute-filler prediction and approaching the Lewis–Nielsen model [113]. This deviation reflects the transition from isolated filler dispersion to connectivity-assisted heat transport dominated by BN–BN conduction pathways [114]. Figure 6b further reveals pronounced anisotropic behavior, where in-plane thermal conductivity is substantially higher than out-of-plane conductivity due to preferential alignment of two-dimensional fillers during processing. While the aligned network enhances lateral heat spreading along the composite plane, through-plane conduction remains limited by interlayer junction resistance and polymer-rich regions [115]. For solar absorber plates, such anisotropic transport can be advantageous because enhanced in-plane conductivity promotes uniform surface temperature distribution and reduces localized overheating.

**Figure 7:**
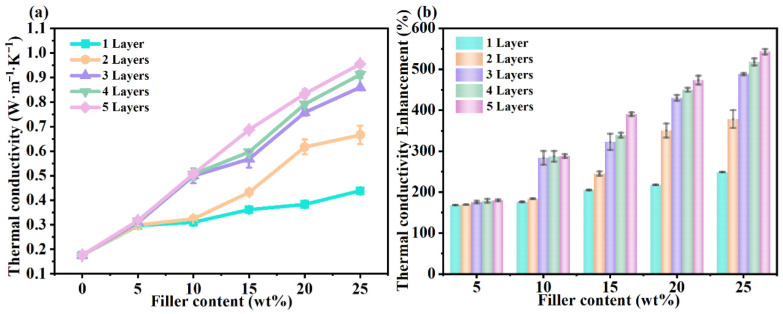
Thermal conductivity evolution in oriented BN multilayer polymer composites. (**a**) Thermal conductivity as a function of filler content for composites with different numbers of aligned BN layers. (**b**) Corresponding conductivity enhancement (%) demonstrating the synergistic effect of multilayer alignment on conductive network formation and heat spreading [116].

A similar structure–property relationship is observed in oriented BN multilayer architectures, as shown in Figure 7. Figure 7a demonstrates that thermal conductivity increases systematically with filler content and that conductivity gains become larger as the number of oriented BN layers increases. This trend confirms that multilayer alignment strengthens continuous in-plane conduction pathways and reduces phonon scattering along the heat-spreading direction. Figure 7b further quantifies this effect through thermal conductivity enhancement percentages, revealing a strong synergistic improvement when multiple oriented layers are stacked. These results indicate that multilayer-oriented platelet architecture provides an effective strategy to enhance absorber heat spreading without requiring excessively high filler loading [116].

Hybrid filler architectures offer an additional strategy for balancing anisotropic heat transport while maintaining high conductivity at moderate filler loading levels [113]. Multi-scale hybrid networks commonly combine ceramic platelets such as BNNS or h-BN [114] with one-dimensional fillers (e.g., CNTs) [115] or small particles (e.g., Al_2_O_3_) [116] or that bridge gaps between aligned platelets. This bridging mechanism reduces contact resistance and promotes three-dimensional conduction pathways while preserving efficient in-plane heat spreading. In solar collector absorbers, hybrid filler systems can also help decouple thermal and optical requirements. Ceramic networks maintain structural stability, compatibility with selective coatings, and limited emissivity impact, while controlled carbon fractions enhance solar absorptance and contribute to heat spreading [114,115,116]. Such hybrid networks are often more robust under thermal cycling because conduction pathways exist across multiple length scales.

In addition to conductivity enhancement, composite microstructure strongly influences thermo-mechanical stability and environmental durability. High filler loading and strong filler networks can reduce the coefficient of thermal expansion and suppress creep deformation, improving dimensional stability under cyclic heating conditions typical of solar collectors [114]. Conversely, poorly dispersed fillers, agglomerates, or voids act as stress concentrators that accelerate crack initiation and degrade thermal pathways during thermal cycling. Aging processes such as UV exposure and thermo-oxidative degradation can further weaken filler–matrix interfaces, gradually reducing conductivity and mechanical integrity over long-term operation [110,111,112,113,114,115,116]. In collector systems, these degradation mechanisms may manifest as increasing temperature gradients, reduced heat transfer efficiency, and eventual delamination of absorber coatings or bonded interfaces. To establish practical structure–property–performance relationships, composite thermal behavior must be interpreted in terms of both anisotropic conductivity and effective heat removal within the collector. High in-plane thermal conductivity promotes uniform absorber surface temperatures, reducing radiative losses and limiting hot spots that accelerate coating degradation [117]. In contrast, higher through-plane conductivity improves heat transfer into fluid channels or bonded tubes, increasing the heat removal factor $F_{R}$ and enhancing useful heat output. Consequently, conductivity enhancement strategies should be evaluated not only based on maximum laboratory conductivity values but also on whether the resulting microstructure provides the conduction orientation required by the collector architecture [114,115,116,117]. Table 4 summarizes how different processing routes influence filler orientation, interfacial quality, anisotropic conductivity, and their implications for collector-level performance.

## 6. Fabrication Techniques and Manufacturing Considerations for Collector-Grade Polymer–Ceramic Hybrid Absorbers

The manufacturability of polymer–ceramic hybrid materials is a critical factor for their practical deployment in solar thermal collectors. Absorber components must be produced at scale while maintaining consistent thermal performance, dimensional stability, and durability under outdoor conditions [125]. In contrast to laboratory-scale composite studies, collector absorbers require strict control of thickness uniformity, tube–absorber bonding quality, surface finish for selective coatings, and resistance to leakage or delamination during thermal cycling. Consequently, fabrication routes must be considered as a collector-level design parameter, since processing methods directly influence filler dispersion, orientation anisotropy, interfacial bonding, porosity, and the reproducibility of thermal conductivity enhancement. Melt processing techniques, particularly extrusion and injection molding, are widely used in industrial polymer manufacturing and therefore represent attractive routes for large-scale production of collector absorber components. Extrusion is especially suitable for producing absorber plates with integrated flow channels, where continuous profiles can be manufactured with tailored cross-sections for heat collection and fluid transport [117]. In polymer–ceramic hybrids, extrusion induces shear-driven alignment of platelet fillers such as boron nitride and graphite, resulting in enhanced in-plane thermal conductivity that supports lateral heat spreading across absorber plates. However, this alignment can reduce through-plane conductivity and create orientation gradients across the thickness, which may limit heat transfer into bonded tubes unless additional design measures such as conductive interlayers or hybrid filler networks are employed. Injection molding enables the fabrication of complex geometries including manifolds, connectors, and integrated absorber modules. Nevertheless, high filler loading can introduce weld-line defects, warpage, and nonuniform filler orientation, potentially affecting dimensional accuracy and long-term sealing reliability. Compression molding and hot pressing remain valuable fabrication routes when high filler loading and low void content are required. These methods enable densification and improved particle contact, thereby reducing interfacial voids and lowering thermal resistance [118]. Hot pressing is particularly effective for platelet-based filler systems, producing aligned laminar structures with high in-plane thermal conductivity. In collector absorber designs, such heat-spreader layers can be combined with polymer channel structures through lamination or bonding, creating hybrid architectures where one layer enhances heat spreading while another provides fluid transport and mechanical support. However, compression-based processes typically have limited capability for complex geometries and often require additional joining steps to integrate tubing or manifolds.

Solution-based processing and in situ polymerization techniques offer improved control over filler dispersion and interfacial bonding, particularly for nanoscale fillers such as boron nitride nanosheets, graphene, and carbon nanotubes. In situ polymerization can produce strong filler–matrix interphases that reduce interfacial thermal resistance and enhance stability under thermal cycling [120]. For collector-grade absorber components, these approaches may enable high-performance materials with improved conductivity retention and mechanical durability. However, challenges related to solvent removal, curing control, and scale-up must be carefully managed to avoid microvoid formation and ensure stable adhesion of selective coatings. Additive manufacturing methods, particularly fused filament fabrication (FFF/FDM), have recently been explored for fabricating customized absorber geometries and complex flow-channel structures [126,127]. In polymer–ceramic composites, additive manufacturing can enable tailored anisotropic conduction by controlling print orientation and layer stacking [128]. However, porosity and weak interlayer bonding frequently reduce through-plane thermal conductivity and compromise leakage resistance, which remain critical limitations for practical collector deployment. As a result, additive manufacturing currently appears more suitable for prototyping and design optimization rather than large-scale commercial absorber production. In addition to bulk fabrication, joining and assembly processes play an important role in determining collector performance. Tube–absorber bonding interfaces, adhesive joints, and lamination layers can introduce significant thermal contact resistance even when high-conductivity composites are used. Interface engineering strategies such as thermally conductive adhesives, bonding films, co-molding, and mechanical interlocking designs are therefore essential to minimize heat-transfer losses and maintain reliability during thermal cycling [124]. Surface finish is also important because polymer–ceramic substrates must be compatible with solar selective coatings, which require stable adhesion under ultraviolet exposure, humidity cycling, and temperature fluctuations.

For practical implementation in solar collectors, fabrication routes must be evaluated not only by achievable composite conductivity but also by scalability, geometry flexibility, anisotropy control, void suppression capability, and compatibility with collector assembly processes. Processing methods strongly influence filler network formation and therefore determine the balance between in-plane heat spreading and through-plane heat transfer into fluid channels. Consequently, manufacturing strategy directly affects collector efficiency, stagnation tolerance, and long-term reliability. Table 5 further compares the major fabrication techniques in terms of microstructural characteristics, collector advantages, and manufacturing limitations, providing guidance for selecting processing routes suitable for large-scale solar collector deployment.

## 7. Durability, Outdoor Aging, and Reliability of Polymer–Ceramic Hybrid Absorbers

The long-term durability of absorber components is a critical requirement for solar thermal collectors, which are typically expected to operate for service lifetimes of 10–25 years under continuous outdoor exposure. For polymer–ceramic hybrid absorbers, durability determines whether improvements in thermal conductivity and optical performance can be maintained during real operation. Although laboratory studies often demonstrate significant conductivity enhancement, the practical viability of these materials depends on the stability of filler networks, resistance of the polymer matrix to environmental degradation, retention of coating adhesion, and reliability of bonded interfaces during repeated thermal cycling. Therefore, collector-grade evaluation must consider time-dependent degradation processes that influence thermal transport, optical performance, and structural integrity. Ultraviolet irradiation represents one of the most significant environmental stressors for polymer-based absorber systems [136]. UV exposure can induce photo-oxidation, polymer chain scission, and progressive embrittlement, leading to surface cracking, discoloration, and reduced mechanical toughness. These changes may accelerate crack propagation during thermal cycling and compromise coating stability. In addition, UV aging can weaken filler–matrix interfaces, increasing interfacial thermal resistance and reducing effective heat-conduction pathways. The severity of UV degradation depends strongly on polymer chemistry, stabilizer formulation, and absorber architecture, particularly whether the polymer substrate is directly exposed to sunlight or protected by glazing or coatings. Ceramic fillers such as TiO_2_ and ZnO may provide beneficial UV-shielding effects by reducing radiation penetration into the polymer matrix; however, their optical scattering behavior must be carefully controlled to avoid reducing solar absorptance of the absorber surface [136]. Consequently, UV durability assessment should include both mechanical integrity and retention of optical properties.

Moisture exposure and humidity cycling present additional durability challenges in solar collectors, particularly under conditions involving daily temperature fluctuations and condensation. Moisture ingress can lead to swelling of the polymer matrix, hydrolysis in susceptible polymers, and degradation of filler–matrix interfaces, especially when hydrophilic fillers attract water at the interface. Over time, these processes may cause interfacial debonding, void formation, and reduced thermal conductivity. Moisture-related effects are especially critical for absorbers that incorporate bonded tubes or lamination interfaces, where swelling and CTE mismatch can generate stresses that promote delamination [137]. Ceramic fillers often improve moisture resistance by increasing composite stiffness and limiting matrix mobility; however, certain fillers such as aluminum nitride may require careful moisture control due to hydrolysis sensitivity [138].

Thermal aging and thermo-oxidative degradation are particularly relevant under collector operating temperatures and stagnation conditions. Prolonged exposure to elevated temperature in the presence of oxygen can cause oxidation of polymer chains, resulting in chain scission, changes in crystallinity, and reduced mechanical ductility. These processes may weaken filler networks and increase the likelihood of microcrack formation during subsequent thermal cycling [136,137]. Thermo-oxidative degradation is often accelerated in localized hot spots, which may occur in absorber designs with insufficient heat spreading or poor thermal contact with fluid channels. Consequently, improving in-plane thermal conductivity and ensuring efficient heat transfer to fluid paths are important strategies for limiting peak temperatures and enhancing durability. High-temperature matrices such as PPS, PEEK, and polyimides offer improved resistance to thermal degradation, whereas commodity polymers require stabilization strategies and careful collector design to avoid creep or softening during stagnation [138].

Thermal cycling represents one of the most severe mechanical stresses encountered in solar thermal collectors. Daily and seasonal temperature fluctuations induce repeated expansion and contraction, and polymer absorbers may experience fatigue damage due to their relatively high coefficient of thermal expansion compared with metallic tubes and coatings [139]. When absorber plates are bonded to metal tubes or coated with selective surfaces, cyclic mismatch stresses can lead to cracking or delamination, increasing thermal contact resistance and reducing heat transfer efficiency. Repeated cycling may also alter filler orientation or disrupt conductive networks if the polymer matrix softens near its glass transition temperature [140]. Hybrid filler architectures can improve cycling stability by providing multi-scale conduction pathways that remain functional even when localized damage occurs. In addition, freeze–thaw cycles in cold climates may introduce further stresses due to moisture expansion, highlighting the need for adequate sealing and moisture-barrier strategies.

The durability of selective absorber coatings and coating–substrate adhesion is equally important because collector efficiency is highly sensitive to optical performance. Even if thermal conductivity remains stable, degradation of the selective coating can reduce solar absorptance or increase thermal emittance, leading to efficiency losses [141]. Polymer–ceramic hybrid substrates should therefore provide stable surface chemistry, appropriate roughness, and sufficient stiffness to maintain coating adhesion during thermal cycling. Ceramic fillers such as boron nitride, alumina, and silicon carbide often improve coating compatibility by increasing surface hardness and dimensional stability, while carbon-rich surfaces may require intermediate layers to ensure reliable coating adhesion [139]. Durability evaluation should therefore include adhesion testing and optical performance monitoring following environmental exposure. To ensure consistent comparison between studies and facilitate translation to collector applications, durability assessment should follow standardized accelerated aging protocols that replicate the major environmental stress modes experienced by solar collectors. These tests should report not only exposure conditions but also retention of key properties after aging, including directional thermal conductivity, mechanical integrity, coating adhesion, and optical absorptance/emittance. Conductivity retention is particularly important because polymer–ceramic hybrids may initially exhibit high thermal conductivity that gradually declines due to interfacial degradation or matrix cracking. Similarly, optical property retention is essential for absorber systems, as changes in solar absorptance or thermal emittance directly affect collector efficiency. Table 6 summarizes recommended accelerated aging protocols and reporting metrics that can support consistent qualification of polymer–ceramic hybrid absorber materials for solar thermal collector applications.

## 8. Comparative Synthesis and Collector-Ready Performance Evidence

Performance data for thermally conductive polymer–ceramic hybrid composites remain difficult to compare across the literature because studies employ different filler morphologies, loading definitions (wt% versus vol%), dispersion protocols, processing routes, and thermal conductivity measurement techniques. As a result, reported conductivity values vary widely even for nominally similar composite systems. Another limitation is incomplete reporting of conductivity direction. Many studies provide only a single conductivity value without clarifying whether it represents in-plane or through-plane transport, although platelet fillers such as boron nitride, graphite, and graphene often produce highly anisotropic composites. Because absorber plates primarily require efficient in-plane heat spreading while tube or channel interfaces require adequate through-plane conduction, directional conductivity reporting is essential for translating laboratory results into collector design. Therefore, collector-oriented comparison of materials should include conductivity directionality, measurement method, fabrication route, test temperature, and durability evaluation. From a collector performance perspective, conductivity enhancement is beneficial only when it reduces absorber temperature gradients and improves heat transfer to the working fluid. Platelet-based boron nitride networks consistently deliver high in-plane conductivity due to strong filler alignment and high intrinsic conductivity, making them effective heat-spreading materials for absorber plates. However, these networks frequently exhibit limited through-plane conductivity, which may restrict heat transfer into tubes or channels unless hybrid filler architectures or interface engineering strategies are implemented. Carbon-based fillers such as graphite and graphene can simultaneously enhance thermal transport and solar absorption, but their application in collector absorbers requires careful control of thermal emittance and optical stability under ultraviolet exposure and stagnation conditions. Hybrid ceramic–carbon architectures offer a balanced strategy by combining thermal conductivity enhancement with tunable optical response and improved coating compatibility when carbon content is carefully controlled. Polymer matrix selection also plays a critical role in maintaining long-term performance under collector operating conditions. Commodity polymers enable cost-effective processing but may exhibit creep deformation and accelerated aging at elevated temperatures, limiting stagnation tolerance. High-temperature matrices such as PPS, PEEK, and polyimides provide improved thermal stability and better preservation of filler network integrity during thermal cycling [149]. Thermosetting matrices may further improve dimensional stability and coating adhesion, although their brittleness under repeated thermal loading must be addressed through suitable toughening strategies [150]. Several studies report that composites lacking appropriate interphase engineering experience conductivity degradation after aging due to filler–matrix debonding, microcrack formation, and increased interfacial thermal resistance [151]. Consequently, collector-grade evaluation should emphasize conductivity retention, coating adhesion stability, and optical property retention rather than only reporting peak conductivity values measured for as-fabricated materials. To facilitate comparison across studies, Table 7 summarizes representative polymer–ceramic hybrid composites that demonstrate collector-relevant thermal performance together with durability evaluation. The compiled evidence highlights that conductivity retention after environmental aging is a critical metric for assessing real absorber suitability.

Comparative evaluation of the most promising studies indicates that collector-compatible polymer–ceramic hybrids typically rely on three design principles: (i) formation of connected filler pathways that provide stable conductivity enhancement at practical filler loading levels [164]; (ii) interphase engineering (e.g., coupling agents or surface functionalization) to reduce interfacial thermal resistance and prevent conductivity decay [165]; and (iii) processing strategies that control filler orientation and suppress porosity, since voids and microcracks rapidly degrade both thermal transport and mechanical reliability during cycling [166]. Platelet-dominant ceramic systems, particularly boron nitride-based composites, are highly effective for in-plane heat spreading and generally provide good chemical stability and coating compatibility [167,168]. Hybrid ceramic–carbon networks can further enhance thermal transport and solar absorption [94,169]; however, emissivity control and optical stability remain important design considerations. Multilayer absorber architectures provide an additional strategy by separating functions: a ceramic-rich heat-spreading layer provides thermal conduction, while a thin selective coating governs optical performance ($\alpha_{solar}$ and $\varepsilon_{thermal}$), reducing dependence on carbon-rich bulk composites [170,171]. For practical collector implementation, composite strategies should be grouped according to absorber component function rather than material class alone. Table 8 therefore summarizes the most effective composite families according to their role within the collector system, linking material design strategies to functional requirements. In addition to component-level optimization, material selection must also consider trade-offs between thermal performance, optical behavior, durability, and manufacturing feasibility. Table 9 presents a simplified decision matrix that links common polymer–filler systems to collector requirements and highlights the primary trade-offs governing absorber design.

Overall, current evidence indicates that ceramic-dominant conduction networks, particularly boron nitride-based systems, offer the most balanced combination of thermal conductivity enhancement, coating compatibility, and long-term durability for solar thermal absorber components [181]. Carbon-rich systems provide strong solar absorption but require careful surface engineering to control emissivity and maintain stability under outdoor exposure. Hybrid ceramic–carbon networks and multilayer absorber architectures appear particularly promising because they allow for simultaneous optimization of thermal transport, optical selectivity, and environmental durability. Future studies should prioritize standardized reporting of directional conductivity, temperature-dependent behavior, and property retention after accelerated aging protocols to enable reliable comparison across materials systems and facilitate translation of laboratory-scale composite developments into collector-ready absorber technologies.

## 9. Challenges, Research Gaps, and Future Roadmap for Collector-Grade Polymer–Ceramic Hybrid Absorbers

Despite significant progress in thermally conductive polymer composites, the translation of polymer–ceramic hybrid materials into reliable absorber components for solar thermal collectors remains constrained by coupled material, component, and system-level challenges [182]. Many studies report impressive thermal conductivity enhancement under laboratory conditions but lack validation under collector-relevant exposure environments, including ultraviolet irradiation, humidity cycling, thermal cycling, and stagnation events [183]. In addition, comparison across studies is often hindered by inconsistent reporting of conductivity direction, measurement conditions, and processing methods, which limits robust synthesis and prevents identification of scalable composite architectures [184]. Addressing these gaps is necessary to transition polymer–ceramic hybrids from laboratory demonstrations to collector-grade technologies. One of the primary challenges is the limited connection between material-level metrics and collector-level performance outcomes [185]. Thermal conductivity enhancement alone does not guarantee collector efficiency improvement, which depends on absorber geometry, heat spreading, tube coupling, bonding interfaces, and fluid flow distribution [186]. Many studies report composite conductivity values without evaluating their influence on the heat-removal factor $F_{R}$, absorber temperature uniformity, or stagnation temperature mitigation [8]. Future research should therefore incorporate component-level validation, including thermal imaging of absorber temperature distribution, heat-transfer measurements in channel structures, and quantification of thermal resistance at tube-bonding interfaces. Such experiments would provide stronger evidence that microstructure-engineered composites deliver meaningful collector-level performance improvements.

Optical selectivity and optical stability remain additional challenges for polymer-based absorber systems [187]. Efficient collectors require high solar absorptance combined with low thermal emittance, yet polymer substrates do not inherently provide selective absorber behavior [14]. Carbon fillers can increase absorptance but may also increase thermal emittance unless selective surface engineering is applied [188]. Ceramic fillers are generally optically stable but require selective coatings to achieve high absorptance [189]. Consequently, compatibility between polymer–ceramic hybrid substrates and selective absorber coatings is an important research area. Future studies should emphasize coating–substrate interface engineering, including surface functionalization, primer layers, graded interphases, and controlled roughness, to maintain optical performance and coating adhesion during long-term environmental exposure.

Interfacial thermal resistance is another critical limitation in polymer absorber systems [190]. Even when the composite material exhibits high intrinsic thermal conductivity, contact resistance at interfaces such as between absorber plates and tubes or between laminated layers may dominate the overall heat-transfer resistance [191]. Polymer substrates are particularly susceptible to this issue because their lower stiffness and higher coefficient of thermal expansion can promote micro-gaps or delamination during thermal cycling [192]. Future research should therefore integrate interface engineering strategies into absorber design, including thermally conductive adhesives, conductive interlayers, mechanical interlocking geometries, and co-molded tube-coupling structures. Ensuring interface durability during repeated thermal cycling is essential for maintaining long-term collector efficiency.

Long-term outdoor durability remains the most decisive challenge for collector-grade implementation [193]. Environmental stressors such as ultraviolet exposure, moisture ingress, thermo-oxidative aging, and thermal cycling can progressively degrade polymer matrices and filler networks. In many published studies, aging tests are either absent or insufficiently standardized, and retention of thermal or optical properties is rarely reported quantitatively. Future development of polymer–ceramic absorber materials therefore requires standardized durability testing protocols that include UV weathering, humidity exposure, stagnation simulation, and thermal cycling, together with reporting of directional conductivity retention, coating adhesion, and optical *α*/ε stability after aging [194]. Combined aging tests are particularly important because synergistic degradation mechanisms often accelerate performance loss beyond single-factor exposures. In addition, polymer matrix selection must account for stagnation tolerance to avoid softening or creep near the glass-transition temperature [195]. Manufacturing scalability and quality control represent another important research gap. Many high-performance composites rely on fabrication routes such as solution casting or laboratory hot pressing, which may not easily scale industrial absorber production. High filler loading can increase melt viscosity, complicate extrusion or injection molding, and introduce voids that degrade conductivity and coating adhesion. Practical collector adoption therefore requires composite formulations compatible with scalable processing methods such as extrusion, compression molding, and lamination. Improved dispersion strategies and process control are also necessary to minimize filler aggregation and porosity while maintaining reproducible conductivity enhancement.

Looking forward, the most promising pathway toward collector-grade polymer–ceramic hybrid absorbers lies in engineered multifunctional architectures rather than single-layer bulk composites. Multilayer absorber designs can separate thermal conduction and optical selectivity functions, enabling ceramic-rich conduction layers for heat spreading combined with thin selective coatings that control absorptance and emittance. Hybrid ceramic–carbon networks may further enhance thermal transport while maintaining optical performance when carbon content is carefully optimized. To accelerate technological maturity, future research should also emphasize technology-readiness-level (TRL) validation through absorber module testing under outdoor conditions, including stagnation events and long-term exposure. Such module-level demonstrations are essential to bridge the gap between laboratory material development and commercial solar collector deployment. Table 10 summarizes the key challenges, research gaps, and recommended research directions for advancing polymer–ceramic hybrid materials toward collector-grade solar thermal absorber applications.

## 10. Conclusions

Polymer–ceramic hybrid composites have emerged as promising candidates for lightweight solar thermal absorber components due to their potential to combine corrosion resistance, reduced weight, and scalable manufacturability with enhanced thermal transport. However, successful implementation in solar thermal collectors requires optimization beyond simple conductivity enhancement. This review has presented a collector-centered evaluation of polymer–ceramic hybrid materials, emphasizing the coupled roles of thermal transport, optical selectivity, durability, and manufacturing readiness in determining absorber performance. Thermally conductive fillers such as boron nitride, aluminum nitride, silicon carbide, and carbon-based materials enable the formation of conductive pathways that significantly improve heat spreading within polymer matrices. Among these systems, platelet-based ceramic networks—particularly boron nitride—consistently provide effective in-plane thermal conductivity enhancement while maintaining chemical stability and compatibility with selective coatings. Hybrid filler architecture combining ceramic and carbon phases can further enhance thermal transport and solar absorption, although careful design is required to avoid increases in thermal emittance or long-term optical instability. At the same time, polymer matrix selection plays a decisive role in determining thermal stability, resistance to environmental degradation, and retention of conductive network integrity during long-term operation.

Microstructure engineering and processing routes strongly influence the resulting thermal performance and durability of polymer–ceramic hybrid absorbers. Filler alignment, dispersion quality, and interfacial bonding determine the balance between in-plane heat spreading and through-plane conduction to fluid channels. Manufacturing strategies such as extrusion, compression molding, lamination, and co-molding must therefore be integrated with composite design to ensure scalable production and consistent thermal performance. In addition, interface engineering at tube–absorber joints and multilayer interfaces is essential to minimize contact resistance and maintain collector efficiency. Durability considerations remain central to the deployment of polymer–ceramic absorbers in real solar collector systems. Environmental stressors including ultraviolet exposure, humidity cycling, thermo-oxidative aging, and repeated thermal cycling can progressively degrade polymer matrices and filler networks if materials are not carefully designed and stabilized. Reliable collector-grade materials must therefore demonstrate long-term retention of thermal conductivity, coating adhesion, and optical performance under realistic aging conditions.

Comparative analysis of available performance data suggests that the most promising absorber architecture combines ceramic-dominant conduction networks with optimized interphase design and scalable processing routes. Multilayer absorber configurations, in which heat-spreading and optical selectivity functions are separated into different layers, represent an especially attractive pathway for balancing thermal transport, optical performance, and durability. Future research should increasingly focus on bridging the gap between material-level characterization and collector-level validation. Standardized reporting of directional conductivity, temperature-dependent behavior, and property retention after accelerated aging will improve comparability across studies. In addition, component- and module-level demonstrations under realistic operating conditions are essential to confirm that microstructure-engineered composites deliver measurable improvements in collector efficiency and long term reliability. Through such integrated material, processing, and system-level development, polymer–ceramic hybrid absorbers have the potential to enable durable, lightweight, and cost-effective solar thermal collector technologies.

## Figures and Tables

**Table 1:** Key material property targets for polymer–ceramic hybrid solar absorber materials.

Property	Representation	Collector-Grade Target Window	Collector Relevance
**Thermal transport properties**			
In-plane thermal conductivity [36]	(k_in-plane_) (W·m^−1^·K^−1^)	≥5 (preferably ≥10)	Improves lateral heat spreading and temperature uniformity
Through-plane thermal conductivity [37]	(k_perp_) (W·m^−1^·K^−1^)	≥1–2	Enables heat transfer into flow channels or tube interfaces
Thermal diffusivity [38]	*α* (mm^2^·s^−1^)	≥1	Supports rapid heat spreading and reduces hot-spot formation
Specific heat capacity [39]	${(C}_{p})$ (J·g^−1^·K^−1^)	≥1.0 and stable after aging	Moderates temperature rise during transient or stagnation conditions
**Thermo-mechanical stability**			
Density (design constraint) [40]	ρ (g·cm^−3^)	minimized while meeting k/CTE targets	Maintains lightweight collector structures
Coefficient of thermal expansion [41]	CTE (µm·m^−1^·K^−1^)	≤40–60	Reduces mismatch with metal tubes or coatings
Service temperature/thermal stability [42]	°C	≥150 °C (≥200 °C desirable)	Ensures stability during stagnation events
Glass transition/melting margin [43]	Tg/Tm	Tg > maximum operating temperature	Prevents softening and creep deformation
**Optical performance**			
Solar absorptance (final absorber) [44]	$\alpha_{solar}$	≥0.90	Maximizes absorbed solar radiation
Thermal emittance (final absorber) [45]	$\varepsilon_{thermal}$	≤0.20	Reduces radiative heat loss
**Durability and environmental resistance**			
Coating compatibility & adhesion [46]	—	Stable after thermal cycling	Ensures durability of selective coatings
UV/weathering resistance [47]	—	Minimal degradation after UV exposure	Maintains optical and mechanical stability outdoors
Moisture/humidity tolerance [48]	—	Stable after humidity or soak testing	Protects filler–matrix interface and coating adhesion

**Table 2:** Polymer matrices for polymer–ceramic hybrid absorbers: collector suitability overview.

Polymer Matrix	Thermal Capability	Key Advantages	Key Limitations	Suitability
PE (polyethylene) [64]	≤80–90 °C	Low cost, lightweight, easy processing	High creep, UV degradation	Low
PP (polypropylene) [65]	≤90–110 °C	Low density, scalable processing	Creep, UV aging, high CTE	Low
PVC/CPVC [66]	≤90–120 °C	Chemical resistance, low cost	UV degradation, brittleness	Low
ABS/PC blends [67]	≤120 °C	Good stiffness, machinability	UV yellowing, creep	Low
PA (nylon) [68]	≤120–140 °C	High strength, good filler interaction	Moisture absorption, hydrolysis	Low
PBT/PET [69]	≤120–150 °C	Dimensional stability	Hydrolysis risk, UV aging	Moderate
Epoxy (thermoset) [70]	≤150–200 °C	Strong adhesion, stable geometry	Brittleness under cycling	High
Silicone resin [71]	≤200 °C	Excellent thermal and UV stability	Low stiffness	Moderate–High
PPS (polyphenylene sulfide) [72]	≤200 °C	Thermal stability, low creep	Higher cost	High
PEI (polyetherimide) [73]	≤200 °C	High stiffness, dimensional stability	Cost, processing limits	High
PEEK [74]	≤250 °C	Excellent thermal stability	High cost, high processing T	High
PI (polyimide) [75]	≤250 °C	Extreme heat resistance	Complex processing, cost	High
Fluoropolymers (PVDF/PTFE) [76]	variable	Weathering resistance	Lower stiffness, bonding issues	Moderate

**Table 3:** Collector-centered overview of ceramic and carbon fillers used in polymer–ceramic hybrid absorber composites.

Filler	Primary Role in Absorber	Thermal Transport Potential	Optical/Durability Impact	Processing Considerations
Hexagonal boron nitride (h-BN) [96]	Heat spreading filler	High; strong in-plane conduction	Optically neutral; excellent chemical and UV stability	Platelet alignment enhances conductivity; surface treatment improves dispersion
Boron nitride nanosheets (BNNS) [97]	High-performance conduction network	Very high at low loading	Stable optical response; excellent aging stability	Requires exfoliation and dispersion control
Aluminum nitride (AlN) [98]	Through-thickness heat conduction	High	Thermally stable; moisture sensitivity must be controlled	Requires moisture control and coupling agents
Silicon carbide (SiC) [99]	Conductivity + mechanical reinforcement	High	Very stable; may increase absorption depending on grade	Higher density; moderate dispersion complexity
Alumina (Al_2_O_3_) [100]	Mechanical reinforcement + moderate conduction	Moderate	Chemically stable; minimal optical impact	Easy processing but higher loading needed
Titania (TiO_2_) [101]	UV protection and optical tuning	Low–moderate	Strong UV shielding; improves weathering resistance	Mainly used as stabilizer or pigment
Zinc oxide (ZnO) [102]	UV shielding and optical modification	Low–moderate	Stable; provides UV protection	Loading must be optimized for optical balance
Graphite (microflake) [103]	Heat spreading + absorption enhancement	High; strong in-plane conduction	Strong solar absorption but may increase emissivity	Alignment increases anisotropy
Graphene nanoplatelets (GNP) [104]	Percolated conduction networks	Very high	High solar absorptance; emissivity control needed	Agglomeration risk; functionalization recommended
Carbon nanotubes (CNTs) [105]	Percolation network at low loading	High	Strong absorption; emissivity increases possible	Dispersion challenges; viscosity increase
Carbon black [106]	Optical absorption pigment	Low–moderate	Very high absorptance; emissivity may increase	Easy processing; widely used pigment
Hybrid ceramic + carbon systems [107]	Balanced thermal and optical design	Very high potential	Tunable absorptance/emittance with good durability	Requires optimization of filler ratios

**Table 4:** Processing–microstructure–property mapping for polymer–ceramic hybrid absorbers.

Processing Route	Microstructural Effect	Anisotropy	Effect on Conductivity	Collector Relevance
Melt extrusion [117]	Platelet alignment along melt flow direction	High	Strong increase in in-plane conductivity; moderate increase in through-plane conductivity	Enhances lateral heat spreading across absorber plates
Compression molding/hot pressing [118]	Dense packing with reduced void formation	Moderate to high	Significant improvement in in-plane conductivity with moderate improvement in through-plane conductivity	Promotes uniform absorber temperature and improved durability
Injection molding [119]	Flow-induced orientation gradients across part thickness	High but nonuniform	Localized enhancement in in-plane conductivity with limited through-plane transport	Suitable for scalable manufacturing of absorber components
Solution casting [120]	Improved dispersion of nanosheets and platelets	Moderate to high	High in-plane conductivity with moderate through-plane conductivity	Suitable for thin absorber layers or coatings
In situ polymerization [121]	Strong interfacial bonding and uniform filler distribution	Low to moderate	Balanced improvement in both in-plane and through-plane conductivity	Supports long-term conductivity retention and durability
3D printing (FDM/FFF) [122]	Layer-by-layer orientation with possible porosity	High	Directional conductivity; through-plane conductivity often limited due to interlayer resistance	Enable customized absorber geometries
Freeze-casting/templating [123]	Engineered three-dimensional filler networks	Designed anisotropy	Tailored directional conductivity depending on network structure	Suitable for advanced high-performance absorber designs
Hybrid multilayer lamination [124]	Layered structures combining functional materials	Designed anisotropy	High in-plane conductivity with tunable through-plane conductivity	Enables separation of optical and thermal functions

**Table 5:** Collector-centered comparison of fabrication methods for polymer–ceramic hybrid absorber components.

Fabrication Technique	Typical Absorber Components	Processing Scale	Collector Advantages	Key Limitations
Melt extrusion [129]	Absorber plates, channel profiles	Industrial, continuous processing	Scalable production; good heat spreading in absorber plates	Limited through-plane conductivity; orientation gradients
Injection molding [130]	Manifolds, connectors, absorber modules	Industrial mass production	Enables complex geometries; high throughput	Warpage, weld-line defects, nonuniform conductivity
Compression molding [131]	Flat absorber plates, lamination layers	Industrial batch processing	Dense parts with stable thermal performance	Limited capability for complex geometries
Hot pressing [132]	Heat spreader layers	Industrial/laboratory scale	High in-plane conductivity and strong heat spreading	Additional joining required for collector integration
Solution casting [133]	Thin films, coating substrates	Laboratory to pilot scale	Good filler dispersion and coating compatibility	Solvent removal and scale-up challenges
In situ polymerization [134]	High-performance absorber layers	Laboratory to pilot scale	Strong filler–matrix bonding and conductivity stability	Processing complexity and curing control
3D printing (FDM/FFF) [126]	Prototypes, complex channel absorbers	Prototype/emerging manufacturing	Design flexibility and rapid prototyping	Porosity, leakage risk, limited through-plane conductivity
Lamination/co-molding [135]	Multilayer absorbers, tube interfaces	Industrial assembly process	Improved thermal contact and anisotropy control	Bond durability under thermal cycling

**Table 6:** Recommended accelerated aging protocols and reporting metrics for collector-grade polymer–ceramic hybrid absorber qualification.

Exposure Type	Typical Accelerated Test Conditions	Major Degradation Risks	Key Evaluation Metrics	Target Performance Criteria
UV weathering [141]	UV-A/UV-B exposure (500–2000 h) with cyclic irradiation/condensation	Photo-oxidation, embrittlement, discoloration	*α_solar_* retention, surface cracking, coating stability	≤10% drop in *α_solar_*; no severe cracking
Humidity aging [142]	85 °C/85% RH for 500–1000 h	Swelling, hydrolysis, interface debonding	Thermal conductivity retention, adhesion strength	≥90% conductivity retention
Water immersion/soak [143]	Water exposure at RT or 60–90 °C	Moisture diffusion, interface weakening	Mechanical retention, dimensional stability	Stable geometry; ≥85–90% conductivity retention
Thermal aging (oxidative) [144]	120–200 °C exposure in air	Oxidation, embrittlement, creep	Tg changes, modulus retention, crack formation	No severe degradation
Thermal cycling [145]	−20 to 120 °C for 200–1000 cycles	Fatigue cracking, delamination	Adhesion strength, conductivity retention	No interface failure
Stagnation simulation [146]	150–220 °C static or cyclic exposure	Softening, creep deformation	Dimensional stability, conductivity stability	Minimal warpage
Freeze–thaw [147]	Wet cycles −20 to 20 °C	Microcracking, leakage risk	Leak testing, microcrack monitoring	No leakage or structural failure
Combined aging [148]	UV + humidity + thermal cycling	Synergistic degradation	Optical retention (*α*/ε), conductivity retention	Minimal performance loss

**Table 7:** Collector-ready performance evidence for polymer–ceramic hybrid composites.

Composite	Fabrication Route	Aging Condition	Property Retention (%)	Collector Relevance
PPS + BNNS [152]	Hot pressing	Thermal cycling (≥500 cycles)	≥90	Absorber heat spreader
PEEK + h-BN [153]	Compression molding	Thermal aging (150–200 °C)	≥85	High-temperature absorber plate
Epoxy + AlN [154]	Casting + curing	Humidity aging (85 °C/85% RH)	≥85	Tube coupling/interface layer
Epoxy + SiC [155]	Casting + curing	Thermal cycling	≥85	Interface laminate
PP + graphite [156]	Extrusion	UV weathering (≥1000 h)	≥80	Low-temperature absorber sheet
PP + h-BN [157]	Extrusion	Humidity exposure	≥85	Heat-spreading absorber
PA + Al_2_O_3_ [158]	Injection molding	Humidity aging	≥80	Structural absorber parts
Silicone + Al_2_O_3_ [159]	Mixing + curing	UV + thermal aging	≥85	Encapsulation/barrier layer
PVDF + BN [160]	Melt compounding	Moisture exposure	≥85	Weather-resistant absorber plate
PPS + BN + CNT (hybrid) [161]	Melt compounding + pressing	Cycling + thermal aging	≥90	High-performance absorber
PP + BN + carbon black [162]	Extrusion	UV aging	≥80	Absorber with enhanced solar absorptance
Multilayer absorber (ceramic conduction + selective surface) [163]	Lamination/co-molding	Combined aging	≥90	Collector-grade architecture

**Table 8:** Recommended polymer–ceramic composite strategies for different collector components.

Collector Component Role	Key Requirement	Recommended Composite Strategy	Performance Advantage	Design Consideration
Absorber plate heat spreading [172]	High in-plane conductivity	BNNS or h-BN platelet networks	Efficient lateral heat spreading	Limited through-plane conductivity
Channel-wall conduction [173]	Balanced directional conductivity	BN platelets with particle bridges (Al_2_O_3_/AlN)	Improved 3D heat conduction	Higher processing viscosity
Tube/heat exchanger coupling layer [174]	Low thermal contact resistance	Hybrid filler + conductive bonding layer	Reduced interface resistance	Bond durability under cycling
Optical absorption enhancement [175]	High solar absorptance	Graphite or graphene with ceramic stabilizer	Enhanced absorption and conduction	Emissivity control required
Selective coating substrate [176]	Coating adhesion stability	Ceramic-rich surface (BN/Al_2_O_3_)	Improved adhesion and stability	Surface roughness control
Encapsulation/weather barrier [177]	UV and moisture resistance	Silicone or fluoropolymer with ceramic stabilizers	Outdoor durability	Lower stiffness

**Table 9:** Collector-grade decision matrix linking polymer–filler systems to absorber requirements.

Design	Thermal Benefit	Durability	Manufacturing Scalability	Typical Collector Application
PP/PE + Al_2_O_3_ [178]	Moderate conductivity improvement	Moderate (requires stabilization)	Excellent	Low-temperature polymer absorbers
PP/PE + BN	High in-plane conductivity	High	Excellent	Heat-spreading absorber sheets
PPS/PEEK + BNNS [54]	Very high conductivity potential	Very high	Good	High-temperature absorber plates
Epoxy + AlN/SiC [179]	High through-plane conductivity	High	Moderate	Interface layers and laminates
PP/PPS + graphite/GNP [180]	High conductivity + solar absorption	Moderate–high	Good	Absorbers requiring high *α_solar_*
Hybrid BN + graphene/CNT [11]	Highest conductivity potential	High	Moderate	Advanced collector absorbers

**Table 10:** Key challenges, research gaps, and recommended research directions for collector-grade polymer–ceramic hybrid absorbers.

Challenge/Gap	Collector-Level Impact	Recommended Research Direction	Priority	Technology Implication
Inconsistent reporting of k directionality & methods [196]	Difficult comparison across studies	Report directional conductivity and test conditions	High	Standardization needed
Weak link between k improvement and collector η [197]	Overestimated efficiency gains	Component and module-level testing	High	Required for design validation
Interfacial thermal resistance at tube bonds [110]	Reduced heat transfer efficiency	Conductive adhesives, interlayers, co-molding	Very high	Critical for absorber performance
Optical selectivity (*α*/ε) trade-off [198]	Increased radiative losses	Multilayer absorbers and selective coatings	Very high	Key absorber design challenge
Emissivity increase in carbon-rich systems [199]	Uncertain long-term performance	Standardized aging tests with property retention reporting	Very high	Essential for collector reliability
Durability under UV/humidity/cycling not validated [200]	Warpage and structural instability	High-temperature matrices and CTE control	Very high	Required for long-term operation
Stagnation tolerance and creep [201]	Difficulty in industrial production	Extrusion-compatible composite formulations	High	Barrier to commercialization
Manufacturing scale-up (viscosity/voids) [202]	Optical degradation and delamination	Surface treatments and primer layers	High	Important for optical stability
Coating adhesion on composite substrates [203]	Limits economic viability	Techno-economic evaluation of filler systems	Medium	Important for commercial adoption

## Data Availability

No new data were created or analyzed in this study.
